# Applicability of crAssphage as a performance indicator for viral reduction during activated sludge wastewater treatment

**DOI:** 10.1007/s11356-023-25824-w

**Published:** 2023-02-17

**Authors:** Ibrahim Ahmed Hamza, Sherif Abd-Elmaksoud

**Affiliations:** Environmental Virology Laboratory, Department of Water Pollution Research, National Research Centre, 33 El Buhouth St., Giza, 12622 Dokki Egypt

**Keywords:** crAssphage, Wastewater, Indicator, Enteric viruses, Water quality, qPCR

## Abstract

A major threat to water quality is the discharge of human-derived wastewater, which can cause waterborne illnesses associated with enteric viruses. A poor association exists between fecal indicator bacteria and virus fate in the environment, especially during wastewater treatment. In the current study, the potential of using a novel human gut bacteriophage crAssphage as a wastewater treatment process indicator was evaluated. Using qPCR, influent and effluent wastewater samples of two wastewater treatment plants were analyzed for crAssphage and human viruses including human bocavirus (HBoV), human adenovirus (HAdV), and human polyomavirus (HPyV). All samples were positive for crAssphage. The annual crAssphage concentrations varied between 1.45E + 04 and 2.39E + 08 gc/l in influent samples and from 1.25E + 04 to 7.88E + 06 gc/l in effluent samples. Human viruses concentrations were some orders of magnitude lower than that of crAssphage. Data demonstrated a significant correlation between crAssphage, HAdV, and HPyV during the wastewater treatment process, suggesting that crAssphage and human viral pathogens have similar removal mechanisms. Ultimately, this work concludes that crAssphage could be a performance indicator for viral reduction in the wastewater treatment process.

## Introduction

Waterborne infections continue to have far-reaching public health and socioeconomic consequences in both the developed and developing worlds. WHO estimates that unsafe water, sanitation, and hygiene cause ~2 million deaths annually, mainly related to infectious diarrhea (WHO 2014).

While viral pathogens are found in water, most countries still use classic fecal indicator bacteria (FIB) which have well-known shortcomings, including insufficiently reflecting viral risk to human health. Many reasons contribute to this, including their increased susceptibility to wastewater/water treatment, sensitivity to disinfectants, low tolerance to environmental conditions, and co-occurrence in animal species (Boehm et al. 2018; Harwood et al. 2005; Payment and Locas 2011).

Enteric viruses are the most prevalent causative agents of gastroenteritis worldwide. Over 150 human pathogenic viruses have been detected in watercourses (Fong and Lipp 2005; Rodríguez-Lázaro et al. 2012). Thus, it is not practical to test the water samples for all of the enteric viruses; thus, surrogate indicators are still needed. Viral fecal pollution indicators have previously been suggested, not yet been extensively utilized for regulatory uses.

These previously discovered markers are divided into two categories: human pathogens and bacteriophages. Human pathogens formerly considered as viral water quality indicators include human adenovirus (HAdV), human polyomavirus (HPyV), and Aichi virus 1 (AiV-1) (Albinana-Gimenez et al. 2009; Hamza et al. 2011; Kitajima et al. 2014). These viral indicators have the benefit of being very specific to humans, but they are limited by low and unpredictable quantities in wastewater.

Bacteriophages have also been proposed as indicators of water quality. These phage-based approaches fulfill the criteria for an ideal viral water quality indicator, such as higher concentrations in wastewater than many human pathogenic viruses and rapid and easy culturability than human viral pathogens (Grabow 2001). Limited specificity to human fecal waste and lower concentrations than other recently found viral targets are potential obstacles to the use of coliphage as an indicator (Grabow 2001; Jofre et al. 2016).

CrAssphage was identified by metagenomic analysis and was claimed to be the most prevalent virus in the human gut (Dutilh et al. 2014) before being proven to be globally dispersed (Edwards et al. 2019). It was highly abundant in the USA and Europe compared to Africa and Asia (Stachler and Bibby 2014).

Further metagenomic analysis revealed that crAssphage is highly specific to human fecal material and was proposed for human fecal source identification (Stachler and Bibby 2014). However, previous research has identified crAssphage in seagull, dog, chicken, cat, and cow feces at lower quantities than in human sewage (Ahmed et al. 2018a, 2018b; Stachler and Bibby 2014). Recent studies have also effectively identified crAssphage in various water matrices impacted with human fecal pollution including river water (Ballesté et al. 2019; Farkas et al. 2019), lake (Ahmed et al. 2018b), stormwater (Ahmed et al. 2018a), and seawater (Sala-Comorera et al. 2021; Sangkaew et al. 2021), showing that crAssphage may be used to identify viral contamination by municipal wastewater. The presence of crAssphage in sewage-impacted waters has also been linked to a higher risk for human health (Crank et al. 2019).

Despite the fact that crAssphage has been studied extensively as a human fecal marker, few studies have yet been performed to assess crAssphage as a process indicator in conventional activated sludge wastewater treatment facilities (Tandukar et al. 2020; Wu et al. 2020). Also, to our knowledge, no data are available on crAssphage in the Egyptian environment. Thus, the primary objectives of the present study were to assess crAssphage removal during activated sludge wastewater treatment and the suitability of crAssphage as a viral process indicator. Over 1-year study, the occurrence and abundance of crAssphage in influent and effluent samples of two WWTPs in Greater Cairo were determined. Moreover, its association with human enteric viruses including HAdV, HPyV, and bocaviruses that showed high dissemination in the Egyptian environment before was demonstrated.

HAdV can cause a variety of diseases including gastrointestinal, respiratory, and urinary infections. HAdV is frequently identified in a variety of water matrices (Bofill-Mas et al. 2006; Hamza et al. 2019, 2011; Hewitt et al. 2013; Pina et al. 1998). Thus, it has been considered as an indicator of human fecal contamination in water. HPyV usually does not produce symptoms in healthy people, but it may cause severe infections in immunocompromised people. HPyV is found in wastewater across the world, and several studies have proposed HPyV as a viral fecal contamination indicator (Albinana-Gimenez et al. 2006; Bofill-Mas et al. 2006). HBoV has been isolated from stool samples collected from patients with gastroenteritis and respiratory tract samples (Allander 2008; Rizk et al. 2021; Weissbrich et al. 2006). Also, different studies showed that HBoV was highly abundant in environmental water samples (Blinkova et al. 2009; Hamza et al. 2017).

## Material and methods

### Study sites and sampling

A total of 46 sewage samples were collected anonymously from two wastewater treatment facilities: WWTP-A and WWTP-B, located in Greater Cairo. Samples were taken monthly as grab samples over a one-year study course between 08/2018 and 07/2019. The designed capacities of these WWTPs are 330,000 m^3^/day for WWTP-A and 600,000 m^3^/day for WWTP-B. The populations served by the WWTPs are approximately 1,320,000 for WWTP-A and 2,200,000 for WWTP-B. Activated sludge is implemented in all WWTPs as a secondary treatment process. Five-liter samples were collected from both the influent and effluent. Samples were collected in sterile bottles and transported within 1 h to the laboratory for analysis.

#### Virus concentration

Virus concentration was performed employing the virus adsorption elution method reported earlier by USEPA (2001). In brief, samples were processed by adding a final concentration of 0.05 M MgCl_2_. The pH was then adjusted to 3.5 with 1 N HCl_._. Then samples were filtrated by a negatively charged HA nitrocellulose membrane with 0.45 m pore size and 142 mm diameter. Prior to the viral recovery using 70 ml of organic elution buffer (3% beef extract, 0.05 M glycine, pH 9.4), the membrane was washed with 0.5 mM H_2_SO_4_, pH 4. The eluates were subjected to an organic flocculation technique for viral re-concentration.

#### DNA extraction

Viral DNA was extracted from 200 µl of concentrated suspension using QIAamp DNA Blood Mini Kit (Qiagen, Hilden, Germany) according to the manufacturer’s instructions. Sterile nuclease-free water was included in each set of extractions as a negative control to monitor cross-contamination. Since environmental samples may have PCR inhibitors which can lead to underestimation of viral concentrations, the frequency of positive samples, murine norovirus (MNV), was added to the samples during extraction as an exogenous control to identify the occurrence of PCR inhibition. A comparison of the Ct value of the MNV to that of the negative control showed no inhibitory effect (data not shown).

#### Quantification of viral genome by qPCR

In this current study, HBoVs, HAdV, and HPyV were included as human viruses and crAssphage was tested as an indicator virus.

Table 1 contains a list of all the primers utilized in the current study. The quantification methodology for HBoV-1 targets the NP1 gene, according to Hamza et al. (2009b). The quantification of HBoV-2, -3, and -4 employed a single-sense primer, whereas qPCR for HBoV-2 and -4 used the same antisense primer, according to Kantola et al. (2010). DNA standards of HBoVs were prepared according to Hamza et al. (2017). HAdV qPCR assay was used by Heim et al. (2003), and HPyV qPCR was used according to Biel et al. (2000). The DNA standards of HAdV and HPyV were prepared according to Hamza et al. (2009a). CrAssphage concentrations were determined using the CPQ_56 assay developed by Stachler et al. (2017). TaqMan probe assay was used for the quantification of all viruses except HBoV-2/4, and 3 SYBR green qPCR assay was conducted.

**Table 1:** Nucleotide sequences of primers and probes used in q(RT)PCR assay

Virus	Target gene	Primer name	Sequence (5`–3`)	Size (bp)	Reference
HBoV-1	NP1	NP1-F2421	TGGCAGACAACTCATCACAG	123	Hamza et al. (2009a, b)
		NP1- R2544	TCTTCGAAGCAGTGCAAGAC		
HBoV-2/4	NS1	HBoV234F	GCACTTCCGCATYTCGTCAG	100	Kantola et al. (2010)
		HBoV24R	AGCAGAAAAGGCCATAGTGTCA		
HBoV-3	NS1	HBoV234F	GCACTTCCGCATYTCGTCAG	100	
		HBoV3R	GTGGATTGAAAGCCATAATTTGA		
HPyV	VP1	PV-TMFOR	TCTATTACTAAACACAGCTTACT	223	Biel et al. (2000)
		PV-BACK	GGTGCCAACCTATGGAACAG		
		PV-Probe	[6FAM] TGGAAAGTCTTTAGGGTCTTCTACCTT[BHQ1]		
HAdV	Hexon	AQ1	GCCACGGTGGGGTTTCTAAACTT	132	Heim et al. (2003)
		AQ2	GCCCCAGTGGTCTTACATGCACATC		
		AdV-Probe	[6FAM] TGCACCAGACCCGGGCTCGGT ACTCCGA [BHQ1]		
CPQ_56	orf00024	056F1	CAGAAGTACAAACTCCTAAAAAACGTAGAG	125	Stachler et al. (2017)
		056R1	GATGACCAATAAACAAGCCATTAGC		
		056P1	[FAM]AATAACGATTTACGTGATGTAAC [MGB]		

TaqMan real-time qPCR reactions were performed in a total volume of 20 µl containing 1 × (10 µl) Quantitect probe PCR kit (Qiagen, Hilden, Germany), 0.5 µM for both forward and reverse primers, 0.2 µM Taqman probe, and 2 µl DNA template. The qPCR program was 95 °C for 15 min as the initial activation step for HotStart Taq DNA Polymerase and 45 cycles of 2-step cycling for 15 s at 94 °C and 1 min at 60 °C. HBoV-2/4 and 3 SYBR green assays were conducted using Maxima SYBR Green qPCR Master Mix Kit (Thermo Scientifc). The PCR conditions were 10 min initial denaturation step at 95 °C, 45 cycles of denaturation at 95 °C for 15 s, and annealing extension at 60 °C for 1 min. Amplification was followed by one cycle of melting curve analysis. Dissociation was carried out from 60 to 95 °C with a temperature ramp of 0.05 °C/s. Analysis indicated a melting peak 81.5 °C ± 0.3 °C for HBoV 2/4 and 80 °C ± 0.2 °C for HBoV-3. In order to exclude data of cross-contamination, negative controls (NTC) were included in each run as nuclease-free water. All NTCs were negative throughout the qPCRs. The amplification and data analysis were performed using Rotorgene 6000.

### Statistical analysis

The viral concentrations were expressed as gc/l of wastewater. Kruskal–Wallis test was used for multiple comparison procedures to determine possible significant variations in the concentrations of crAssphage and human enteric viruses. Human viruses concentrations were normalized as the ratios over crAssphage concentrations to evaluate differential fate. Wilcox test was used to compare the ratio of enteric viruses over crAssphage from influents and effluents. Spearman’s rank correlation coefficients (*r*) were calculated between viral concentrations using two-tailed 95% confidence intervals.

## Results

### Detection rates of crAssphage and human viruses

Over a one-year study, all viruses could be detected in the tested wastewater samples at different frequencies (Table 2). HboV-2/4, HBoV-3, and crAssphage were the most frequently detected in influent samples of WWTPs. Influent samples were positive for at least 5 out of six viruses. No clear seasonal pattern was observed for neither the human pathogenic viruses nor the indicators. In effluent samples, there was a slight difference between the detection rates of human enteric viruses, except for HBoV-1; it was only detected in seven and four samples of WWTP-A and B, respectively. CrAssphage was identified in 100% (*n* = 23) of effluent samples (Table 2).

**Table 2:** Prevalence of CrAssphage and human viruses in influent and effluent of wastewater treatment plants

	Plant	No. of positive samples (%)		
		Inlet	Outlet	Total
HBoV-1	WWTP-A	83.3%(10/12)	63.6% (7/11)	73.9% (17/23)
	WWTP-B	81.8% (9/11)	36.3% (4/11)	59% (13/22)
Total		(19/23)	(11/22)	66.6% (30/45)
HBoV-2	WWTP-A	100% (12/12)	100% (11/11)	100% (23/23)
	WWTP-B	100% (11/11)	81.8% (9/11)	90.9% (20/22)
Total		100% (23/23)	90.0% (20/22)	95.5% (43/45)
HBoV-3	WWTP-A	100% (12/12)	91% (10/11)	95.6% (22/23)
	WWTP-B	100% (11/11)	91% (10/11)	95.5% (21/22)
Total		100% (23/23)	90.9% (20/22)	95.5% (43/45)
HPyV	WWTP-A	83.3%(10/12)	81.8% (9/11)	82.6% (19/23)
	WWTP-B	72.7% (8/11)	63.6% (7/11)	68.2% (15/22)
Total		78.2% (18/23)	72.7(16/22)	75.5% (34/45)
HAdV	WWTP-A	75% (9/12)	90.9% (10/11)	82.6% (19/23)
	WWTP-B	90.9% (10/11)	90.9% (10/11)	90.9% (20/22)
Total		82.6% (19/23)	90.9% (20/22)	86.7% (39/45)
CrAssPhage	WWTP-A	100%(12/12)	100% (12/12)	100% (24/24)
	WWTP-B	100% (11/11)	100% (11/11)	100% (22/22)
Total		100% (23/23)	100% (23/23)	100% (46/46)

#### Concentrations of crAssphage and human viruses

**Fig. 1:**
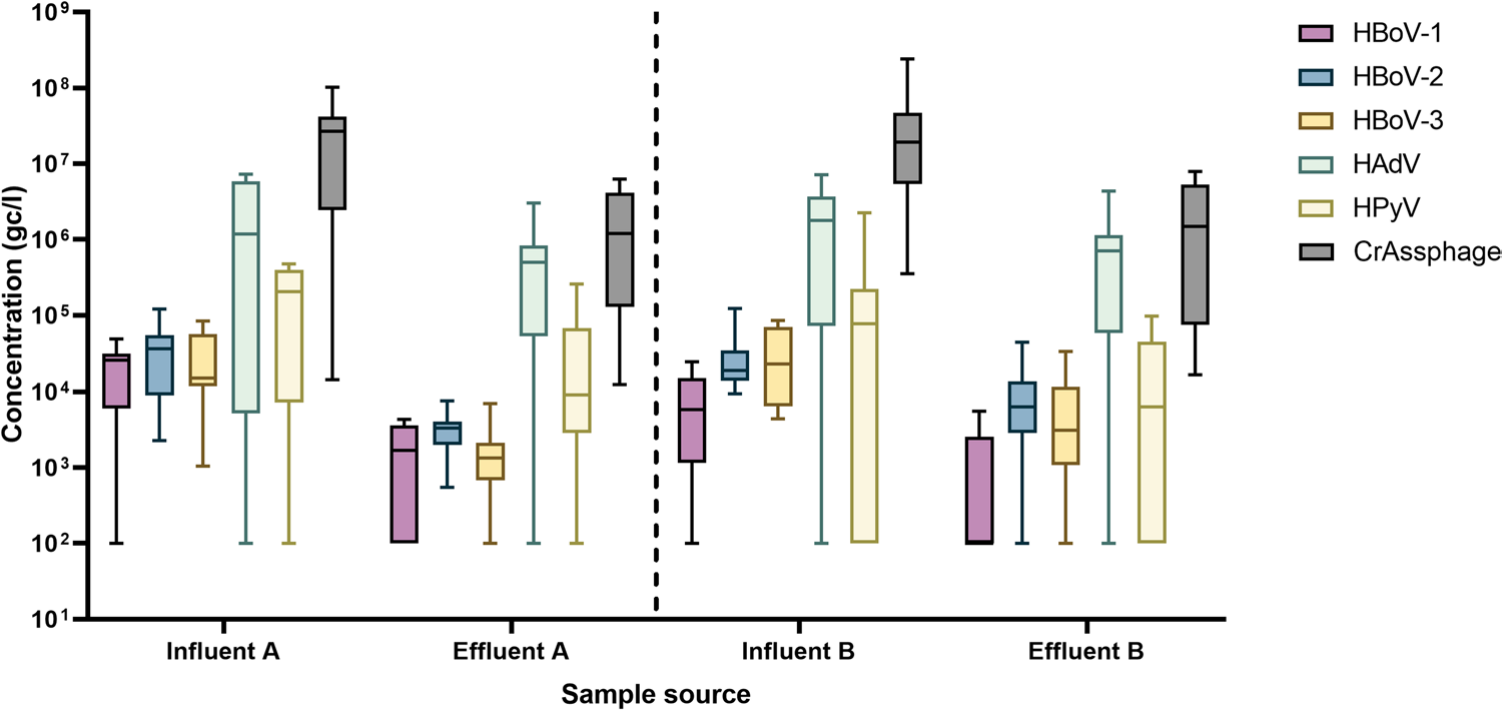
Box plot showing a comparison between viral levels in WWTPs A&B. The inner box lines show the medians, while the outer box lines represent the 25th and 75th percentiles. Whiskers show min–max values. The *x*-axis shows the sample source, and the *y*-axis shows the viral concentration in genome copy number per liter (gc/l)

The concentration of crAssphage in influent samples was significantly higher than those of HAdV, HPyV, and HBoVs (ANOVA), *p* < 0.0001. In wastewater influent samples, the concentration of crAssphage ranged from 1.45E + 04 to 1.02E + 08 gc/l in WWTP-A and from 3.51E + 05 to 2.39E + 08 gc/l in WWTP-B (Fig. 1). Regarding human viruses, HBoVs were detected in effluents wastewater samples at concentration orders of magnitude lower than HAdV and HPyV (Fig. 1). Similarly, in effluent samples of WWTPs, the concentration of crAssphage was significantly higher than HBoVs and HPyV, ranging from 1.25E + 04 to 6.29E + 06 gc/l in WWTP-A and 7.49E + 04 to 7.88E + 06 gc/l for WWTP-B (Kruskal–Wallis test, *P* = 0.0001) (Fig. 1). However, no significant difference between crAssphage concentration and HAdV in effluent wastewater samples was identified.

**Fig. 2:**
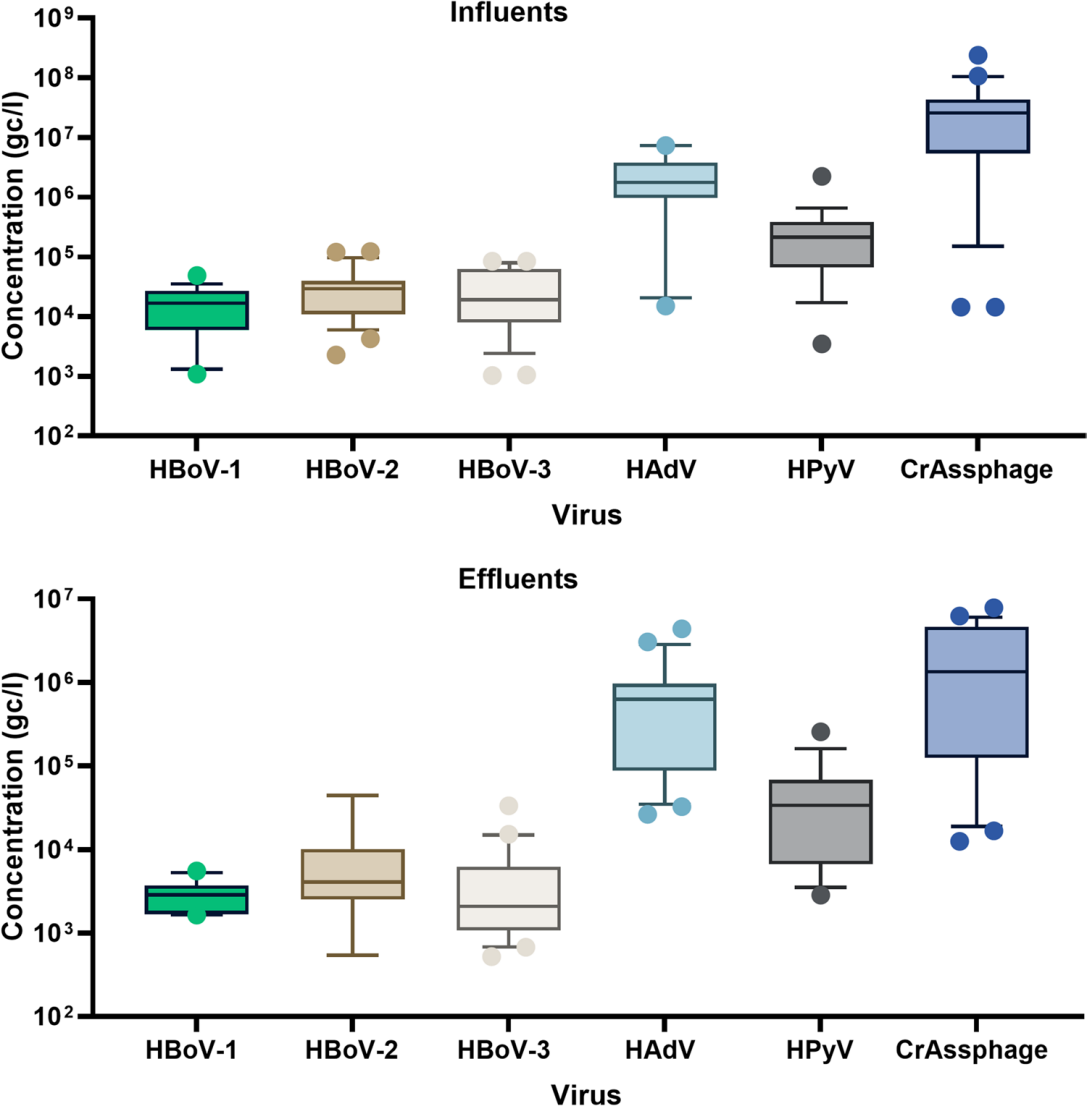
Box plot represents an overall comparison of crAssphage and human viruses in influent and effluent samples from two WWTPs in Cairo, Egypt. The *x*-axis shows the virus type, and the *y*-axis represents the concentrations in genome copy per liter (gc/l). The inner box lines show the medians, while the outer box lines represent the 25th and 75th percentiles

Additionally, in Fig. 2, the overall annual viral concentration is compared between influent and effluent samples of WWTPs. CrAssphage and human enteric viruses concentrations were relatively stable during the study course.

#### Viral reduction during the treatment process

**Fig. 3:**
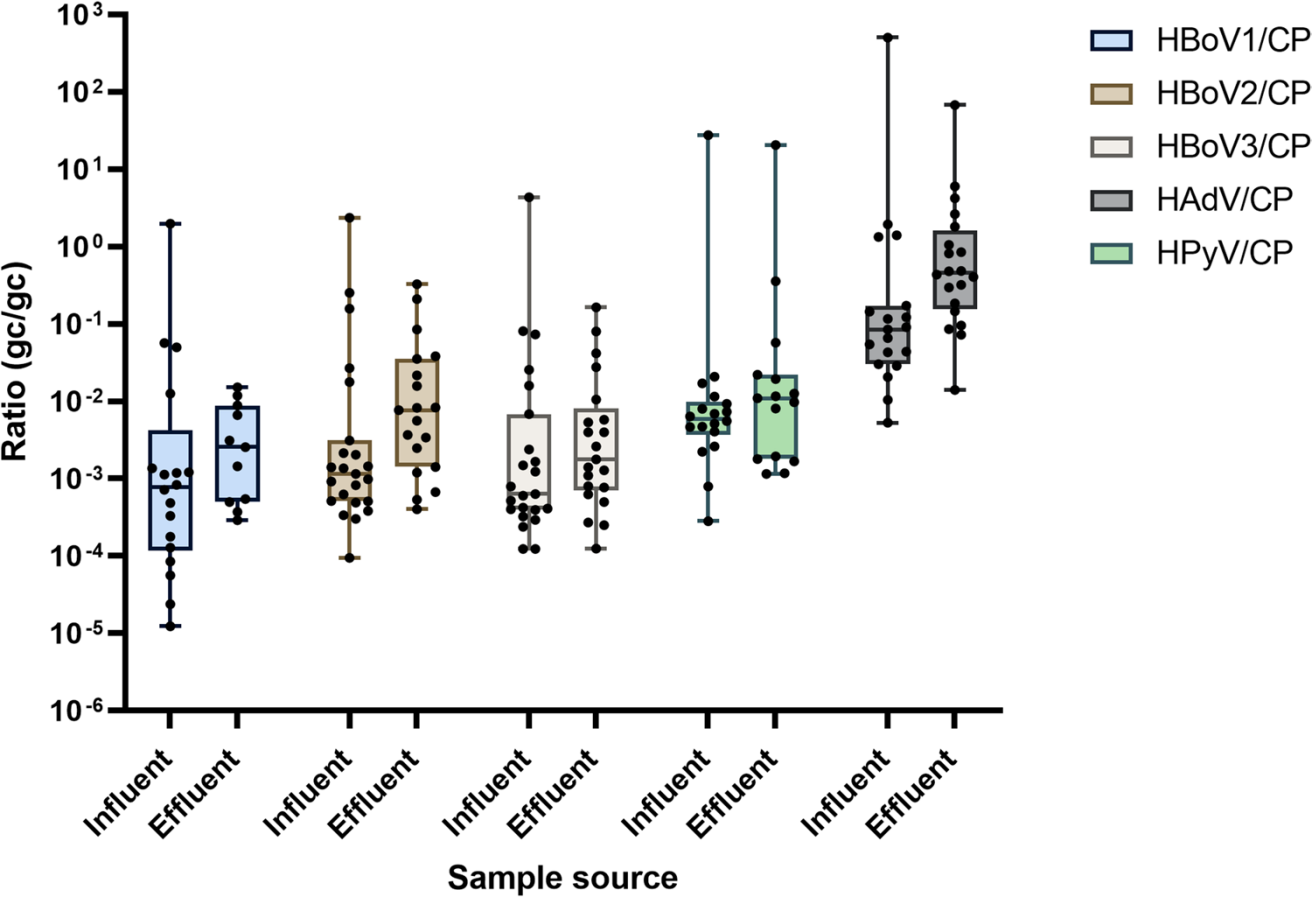
Boxplots of the ratios of human pathogenic viruses concentrations at influent and effluent samples normalized over crAssphage concentrations. The *x*-axis shows the sample source; the *y*-axis shows the ratio of genome copy number gc/gc

The annual mean reduction of all tested viruses was relatively similar, varying between ~ 1 ± 0.64 log_10_ for HBoVs, 0.84 ± 0.5 log_10_ for HAdV, 1.1 ± 0.8log_10_ for HPyV, and 1.32 ± 0.7log_10_ for crAssphage. No significant difference between viral reduction was observed. Figure 3 shows ratios of human pathogenic viruses concentrations at influent and effluent samples, normalized over crAssphage concentrations. The ratio showed a slight increase from influents to effluents. These ratios were used to assess the differences in the fate of crAssphage and other human viruses during the wastewater treatment process. Only samples with both targets within the quantifiable range were considered in pair comparison.

#### CrAssphage correlation with human viruses

Spearman’s rank correlation coefficients (*r*) were determined between human viruses and crAssphage concentrations in influent and effluent wastewater samples. As noted in Table 3, a strong positive correlation (*P* = 0.001) was found between crAssphage and HPyV in influent samples. Also, a significant correlation (*P* < 0.05) was detected between crAssphage and both HAdV and HPyV in the treated samples.

**Table 3:** Spearman correlation between the concentration of crAssphage and human enteric virus

Sample	Virus	*R*	*P*
WWTP-Inlet	CrAssphage vs. HBoV-1	0.18	0.67
	CrAssphage vs. HBoV-2/4	0.22	0.33
	CrAssphage vs. HBoV-3	0.27	0.21
	CrAssphage vs. HPyV	0.68***	**0.001**
	CrAssphage vs. HAdV	0.16	0.4
WWTP-outlet	CrAssphage vs. HBoV-1	0.36	0.09
	CrAssphage vs. HBoV-2/4	0.26	0.23
	CrAssphage vs. HBoV-3	0.40	0.06
	CrAssphage vs. HPyV	0.43*	**0.04**
	CrAssphage vs. HAdV	0.45*	**0.04**

The asterisks indicate the significance level of each pair of data sets (no asterisk: P > 0.05, *p < 0.05; **p < 0.01; ***p < 0.001). Values in bold represent significant associations

## Discussion

No data are available on crAssphage in the Egyptian environment. The primary objectives of the current work were to assess crAssphage reduction during wastewater treatment and its usefulness as a viral process indicator of the treatment process. Thus, targeted research typically selects pathogens that are more relevant to humans or that are more abundant in wastewater. Some of these viruses have been involved in the present study.

Samples were taken from influents and effluents of two WWTPs, and the results of crAssphage genome levels were compared with that of different human enteric viruses. All wastewater samples tested positive for crAssphage (Table 2), with no identifiable seasonal variations. Also, both WWTPs showed relatively the same range of viral concentrations (Fig. 1) due to using the same treatment technology regardless of their treatment capacity. In raw sewage, the annual crAssphage concentrations varied between 1.45E + 04 and 2.39E + 08 gc/l (Fig. 2). The log_10_ concentrations of crAssphage in our study are lower than the previously detected values in Florida, USA (9–10 log_10_gc/l) (Ahmed et al. 2018a), Spain (8.4–9.9log_10_ gc/l) (García‐Aljaro et al. 2017), Japan (10.98–12.03log_10_ gc/l) (Malla et al. 2019), Indiana, USA (8.23 ± 0.36 log_10_ gc/l) (Wu et al. 2020), and UK (5.3–9 log_10_ gc/l) (Farkas et al. 2019). The detected crAssphage concentration in this study is relatively the same as the previously reported in Thailand (5.23–7.19 log_10_ gc/l) (Kongprajug et al. 2019). On the other hand, crAssphage concentrations in effluent samples ranged from 1.25E + 04 to 7.88E + 06 gc/l which is relatively the same range as determined in effluent samples examined by Kongprajug et al. (2019). Whereas others from different geographical areas have reported higher concentrations of crAssphage in effluent samples (Ballesté et al. 2019; Malla et al. 2019; Tandukar et al. 2020).

The difference in crAssphage between different studies could be attributed to the different geographic distribution of viruses, the capacity of WWTPs, and the difference in industrialized lifestyle (Honap et al. 2018; Stachler and Bibby 2014). Moreover, using different concentration techniques, processed water samples, and the quantification method can contribute to the discrepancies in the viral concentrations from different investigations. Additionally, the diversity of crAssphage in the human gut has been recently described (Edwards et al. 2019). It is likely that such natural diversity in crAssphage was not detected by the CPQ56 assay which was designed based on the prototype crAssphage.

A comparison between the level of crAssphage and human viruses showed that in influent and effluent samples, the mean concentration of crAssphage has one order of magnitude higher than HAdV and HPyV and three orders of magnitude higher than HBoVs (Fig. 2). Similar trends have been observed in recent reports. Farkas et al. (2019) estimated that all viruses-positive wastewater samples contained approximately 2 log_10_ higher crAssphage than other enteric viruses such as NoV, AdV, and HPyV. Also, crAssphage was up to 5 orders of magnitude higher than HPyV in wastewater (Stachler et al. 2018). The present data showed no seasonal pattern for human viruses and crAssphage. This finding is consistent with other year-long monitoring investigations that have also revealed the constant existence of crAssphage in treated wastewater without seasonal variations (Crank et al. 2020; Farkas et al. 2019; Wu et al. 2020). Meanwhile, the levels of human enteric viruses may have more variations according to the clinical situation of the population.

In general, the annual mean reduction of all tested viruses between ~ 1 ± 0.64 log_10_ for HBoVs, 0.84 ± 0.5 log_10_ for HAdV, 1.1 ± 0.8 log_10_ for HPyV, and 1.32 ± 0.7 log_10_ for crAssphage. Our results agree with Farkas et al. who found up to 2 log_10_ reduction in crAssphage using activated sludge treatment and lower reduction levels (1 log_10_) by biofilter treatment (Farkas et al. 2019). Tandukar et al. (2020) observed that crAssphage had the greatest removal ratio (3.3 ± 1.0 log_10_) among studied enteric viruses such as HPyV, NoVGII, EV, and AiV. Accordingly, Tandukar et al. (2020) argued that crAssphage cannot be used as an indication of viral reduction throughout wastewater treatment. Another study by Wu et al. (2020) reported that the log_10_ reduction of crAssphage (2.88 ± 0.68) during wastewater treatment was relatively higher than HAdV (2.24 ± 0.53) or HPyV (1.51 ± 0.37). Although crAssphage had a greater initial concentration in the main influent, the variation in removal is likely limited to crAssphage since it was eliminated in a higher fraction than HAdV or HPyV after secondary treatment (Wu et al. 2020).

Ultimately, the log_10_ removal rate of HBoV, HAdV, and HPyV during activated sludge treatment was reported as 0.35–1 log_10_, 0.8–3.7 log_10_, and 1.0–3.7 log_10_, respectively (Hamza et al. 2017, 2011; Kitajima et al. 2014; Sangkaew et al. 2021; Schmitz et al. 2016). While crAssphage log reductions are less variable than that of other viruses, the results suggest that crAssphage has a high potential as a process indicator for pathogenic viral reduction during wastewater treatment.

The ratio of viruses over crAssphage (Fig. 3) has been slightly increased from inlet to outlet samples indicating slightly lower removal of human viruses than crAssphage (Wilcox test, *P* > 0.05). Notably, crAssphage was detected in all samples, and lower detected rates have been identified for other human viruses. Data normalization over crAssphage has been proposed before to assess the performance of the wastewater treatment process. For instance, Wu et al. (2020) reported that ratios of HAdV/CPQ56 and HPyV/CPQ56 increased during secondary treatment, indicating that both viruses were removed relatively smaller than crAssphage. However, both viruses had the same removal mechanism owing to the correlation between crAssphage and HAdV and HPyV.

A correlation between viral human fecal indicators and viral pathogens in wastewater is required to obtain an accurate picture of the viral risk posed by human feces. The present study compared the concentration of crAssphage with HBoVs, HPyV, and HAdV. In influent samples, the co-occurrence analysis between crAssphage and human viruses revealed a strong positive correlation between crAssphage and HPyV. However, HAdV and HPyV correlated with crAssphage in effluent samples (Table 3). This finding is consistent with a report of crAssphage concentration correlating with HPyV and HAdV through a WWTP (Wu et al. 2020). Similarly, Crank et al. (2020) observed a positive correlation between crAssphage and DNA viruses (HPyV, HBoV) in raw sewage samples and no correlation was found between crAssphage and HEV. Although the virus enrichment approach may affect this association, the correlation between crAssphage and HPyV was stable regardless of the concentration method (Crank et al. 2020). Additionally, concentrations of crAssphage in raw wastewater correlated positively with the concentrations of HAdV, HPyV, and NoVGII (*p* < 0.05), suggesting the applicability of crAssphage as a suitable indicator to estimate human enteric virus concentrations in raw wastewater. Likewise, Farkas et al. (2019) found a positive correlation between HPyV and crAssphage in both influent and effluent samples. It should be noted that locality and crAssphage marker selection in qPCR assays were likely to contribute to the observed correlations (Sabar et al. 2022). Future studies should investigate which crAssphage markers correlate well with each water-related pathogen in different locations.

The ideal viral indicator to assess the performance in wastewater should be prevalent at a high concentration in raw sewage and has similar or more persistence in wastewater treatment than the pathogenic viruses of the reduction target. CrAssphage possesses several properties that would make it a potential viral process indicator during wastewater treatment. In raw sewage, it was the most abundant of the fecal markers utilized in the current investigation, making it easier to determine. The virus was more persistent during the treatment process than human viruses enabling the performance assessment. Also, high values of crAssphage could be found in the effluent samples, promoting the evaluation of the treatment process in terms of log reduction. Alternatively, crAssphage meets Bonde’s criteria for an ideal indicator of waterborne pathogens, which include (i) being present when the pathogens are present, (ii) occurring in greater numbers than pathogens, and (iii) being more resistant to disinfectants and to aqueous environments than the pathogens (NASEM 2004).

## Conclusions

The current study aimed to assess crAssphage reduction in WWTPs and to evaluate its usefulness as a viral process indicator during the treatment process. When crAssphage was compared to human viruses, crAssphage was highly abundant in both raw and treated wastewater samples without a significant difference in the removal rate. Importantly, crAssphage is associated with different human viruses in raw and treated wastewater samples. Also, the high co-occurrence and comparable destiny of crAssphage to human viruses such as HAdV and HPyV during the treatment process shows that crAssphage and human viral pathogens have similar removal mechanisms. These findings provide additional evidence of the usefulness crAssphage as a process indicator for wastewater treatment. Additionally, the constant high prevalence, abundance, and association with human pathogenic viruses including HAdV and HPyV in wastewater support its use as a conservative viral indicator of human fecal pollution. Since this study compared the fate of crAssphage and human DNA viruses in WWTPs, further evaluation including RNA viruses should be performed.

## Data Availability

All data generated or analyzed during this study are included in this published article.
